# Utility of Blood Markers for Predicting Outcomes of Fertility Preservation in Patients With Breast Cancer

**DOI:** 10.3389/fendo.2022.803803

**Published:** 2022-02-23

**Authors:** Yeon Hee Hong, Seul Ki Kim, Jung Ryeol Lee, Chang Suk Suh

**Affiliations:** ^1^ Department of Obstetrics and Gynecology, Seoul National University Bundang Hospital, Seongnam, South Korea; ^2^ Department of Obstetrics and Gynecology, Seoul National University College of Medicine, Seoul, South Korea; ^3^ Health Promotion Center, Seoul National University Bundang Hospital, Seongnam, South Korea; ^4^ Department of Surgical Oncology, Sheikh Khalifa Specialty Hospital, Ras Al Khaimah, United Arab Emirates

**Keywords:** blood markers, breast cancer, controlled ovarian stimulation (COS), fertility preservation, mature oocyte

## Abstract

This study aimed to investigate the usability of blood markers for predicting controlled ovarian stimulation (COS) outcomes in patients with breast cancer undergoing fertility preservation (FP). In total, 91 patients with breast cancer who had undergone COS using a letrozole-combined gonadotropin-releasing hormone (GnRH) antagonist protocol before chemotherapy were enrolled retrospectively in a single tertiary hospital. FP outcomes were compared in terms of the mean platelet volume (MPV), MPV/platelet count (PC), neutrophil-to-lymphocyte ratio (NLR), platelet-to-lymphocyte ratio (PLR), and lymphocyte-to-monocyte ratio (LMR). The cutoff values for obtaining 10 or more mature oocytes as favorable prognoses were obtained for each parameter, and the COS outcomes were compared based on the cutoff values. The optimal cutoff levels for MPV and MPV/PC were 10.15 [sensitivity: 90.0%; specificity: 45.1%; AUC: 0.687; 95% CI (0.563, 0.810)] and 0.41 [sensitivity: 65.0%; specificity: 67.6%; AUC: 0.682; 95% CI (0.568, 0.796)], respectively. The oocyte numbers did not significantly differ with respect to the cutoff values of NLR, PLR, and LMR (p > 0.05). However, the total number of acquired and mature oocytes were significantly lower in the group with MPV<10.15 than in that with MPV≥10.15 (8.0 ± 5.1 *vs.* 12.6 ± 9.1, p=0.003; 4.0 ± 3.7 *vs.* 7.3 ± 6.3, p=0.002, respectively). Similarly, considering the cutoff of MPV/PC as 0.41, the low-MPV/PC group showed a significantly lower total oocyte yield than the high-MPV/PC group (9.5 ± 7.1 *vs.* 13.1 ± 9.1, p=0.048), whereas the number of mature oocytes showed similar patterns with no statistical significance (5.3 ± 5.4 *vs.* 7.3 ± 6.1, p=0.092). From logistic regression analysis, age, anti-Müllerian hormone (AMH) level, MPV, and MPV/PC≥0.41 were found to be significant factors for the acquisition of 10 or more MII oocytes (p=0.049, OR: 0.850; p<0.001, OR: 1.622; p=0.018, OR: 3.184; p=0.013, OR: 9.251, respectively). MPV or MPV/PC can be a reliable marker for predicting FP outcome in patients with breast cancer. Protocols to acquire more mature oocytes, such as the dual-trigger approach, could be recommended for patients with breast cancer with MPV<10.15. Furthermore, a higher dose of gonadotropins was considered to obtain more oocytes in patients with MPV/PC<0.41.

## Introduction

Breast cancer is the most common cancer worldwide, as well as in Korea, and accounts for 20% of all cancers in female patients (1). The 5-year survival rate is reported to be 93.2%, second highest after that of thyroid cancer. In 2016, the life expectancy of women was reported to be 85.6 years; therefore, life plans after cancer treatment have also become an important issue. Many young women of childbearing age are diagnosed with breast cancer; therefore, pregnancy, childbirth, and restoration of fertility are important factors for improving the patients’ quality of life after treatment.

Breast cancer treatment may differ depending on the stage and patient’s condition; surgery, chemotherapy, and radiation and hormone therapy are currently the most recommended modalities. In patients receiving chemotherapy, toxicity of the drugs poses a significant threat to the ovaries and uterus. Even if chemotherapy is not used, contraception is recommended when using hormonal inhibitors for 5-10 years. Therefore, for women who want to become pregnant and give birth after treatment, preservation of fertility before gonadotoxic therapy is essential.

Oocyte cryopreservation (OC) and embryo cryopreservation (EC) are established options for fertility preservation (FP). On an average, 10–14 days of ovarian stimulation are required; thus, only one attempt is usually made before surgery or chemotherapy, and one additional attempt after surgery can also be made to achieve fertility. Considering the limited opportunities to preserve fertility before the initiation of cancer treatment, a patient-tailored protocol would be necessary to achieve maximum efficiency within these opportunities.

To increase the efficiency of controlled ovarian stimulation (COS) outcomes, several markers related to prognosis associated with breast cancer, such as hormone receptor profiles [estrogen receptor (ER), progesterone receptor (PR), and human epidermal growth factor receptor 2 (HER2)], and BRCA mutations, have been studied, in addition to conventional ovarian reserve markers, such as anti-Müllerian hormone (AMH), for their correlation with COS outcomes. The studies have shown that triple-negative breast cancer (TNBC) is associated with a poorer rate of acquisition of sufficient mature oocytes compared to non-TNBC (2). BRCA1 mutation carriers show a lower AMH level and a lower rate of acquisition of mature oocytes than BRCA2 carriers or patients with non-BRCA mutations (3). However, since the precise relationships between each marker and COS outcome had not been evaluated in previous investigations, the results are controversial. Moreover, since no other marker has been studied till date, individualization of the FP strategy is difficult. The biggest drawback of the markers that are currently being studied is that they require specific tests, which involve approximately 1 to 2 weeks for breast biopsy result and special staining, and an additional 2 months for confirmation of the BRCA mutation. Results of such tests are difficult to apply directly to COS, making the biomarkers unsuitable as predictive markers. The ER and/or PR hormone receptors are important markers associated with the pathophysiology of breast cancer and prognosis; however, not many studies have evaluated them yet. Therefore, development of an alternative marker that can be easily and quickly tested, and immediately reflect COS outcome, is necessary.

Blood markers can be used as alternative markers in terms of cost-effectiveness. Complete blood cell count (CBC) is very useful in this regard, since it reflects the systemic inflammation and immune response of the host while providing results within 1-2 h. The systemic inflammatory response involves changes in the relative levels of circulating white blood cells (WBCs). The effectiveness of blood cells as disease-markers has already been investigated in previous studies. Inflammation influences cancer progression, and chronic systemic inflammatory responses have been found to be associated with poor outcomes in patients with breast cancer (4). Platelets play an important role in tumor progression and prognosis by interacting with both tumors and host microenvironments. Mean platelet volume (MPV), a platelet indicator, can reflect thrombocytopenia, and high MPV occurs when platelets are lost and thrombopoietin levels are increased to yield new, large platelets (5). In patients with cancer, a low MPV is associated with poor prognosis (6) and is linked to an increased risk of venous thromboembolism (7). Lymphocytes perform immune surveillance against tumors and inhibit tumor progression and metastasis (8, 9). An abundance of tumor-infiltrating lymphocytes in patients with breast cancer is associated with a favorable prognosis (10, 11). Neutrophils are also closely related to the prognosis of breast cancer; higher neutrophil-to-lymphocyte ratio (NLR) and platelet-to-lymphocyte ratio (PLR), calculated on the basis of the number of each cell, are known to be associated with poor survival (12). The lymphocyte-to-monocyte ratio (LMR) has also been reported to reflect the systemic inflammatory status; patients with breast cancer having low LMR showed worse survival than those having high LMR (13).

Ovarian sex steroids (estradiol (E2) or progesterone (P4)) can also affect the immune system. Estrogen and progesterone have been shown to stimulate peripheral blood monocytes (14); it can be postulated that the action or number of blood cells can vary in response to reproductive events (15). There have been several studies on blood markers related to infertility (16–19). Correlations between various inflammatory markers such as MPV, LMR, NLR, and PLR have been analyzed in *in vitro* fertilization (IVF) cycles for patients with infertility. However, no consistent finding has been reported yet. A positive correlation was reported between MPV and pregnancy rate in women with polycystic ovary syndrome; however, no other study reported any correlation between inflammatory markers and pregnancy outcomes. There have been very few studies previously in patients with breast cancer who had undergone COS for FP.

In this single center retrospective study, we evaluated blood markers, such as NLR, PLR, LMR, MPV, and MPV/platelet count (PC), as inexpensive and easily usable potential markers for predicting COS outcomes in patients with breast cancer undergoing FP. With the new markers, we aimed to establish an individualized COS strategy for patients with limited opportunities for FP.

## MaterialS and Methods

### Patients and Enrollment Criteria

All patients with pathologically confirmed breast cancer who underwent COS for FP between September 2012 and April 2021 at Seoul National University Bundang Hospital (SNUBH) were included in the study. All COS cycles before chemotherapy were eligible and conducted using the letrozole-combined gonadotropin-releasing hormone (GnRH) antagonist protocol. For cases involving EC, procedural outcomes up to the final oocyte acquisition were analyzed. Patients with all blood parameters available within 2 months between blood sample collection and COS start were eligible. Cycles with a history of previous chemotherapy before COS, no oocyte acquisition, or cancer recurrence were excluded from the study. Other exclusion criteria included the presence of proven severe infections, hematologic disorders, cardiopulmonary disease, and renal or hepatic disorders. Based on these criteria, 91 patients were finally enrolled in this study. All information was obtained from electronic medical records.

### Prognostic Markers

Tumors were classified into subtypes according to the immunohistochemical expression of receptors. The cut-off values for both ER and PR positivity were >0% positive for tumor cells with nuclear staining. HER2 overexpression was determined by immunohistochemical staining (20). Histological grading was performed based on the degree of differentiation, reflecting the amount of gland formation (cell differentiation), nuclear features (degree of pleomorphism), and mitotic activity, using the Bloom Richardson Grading System (21). The above three factors were reflected in the scoring; grade 1 (well differentiated) when the total score was 3-5; grade 2 (moderately differentiated) when the total score was 6-7; and grade 3 (poorly differentiated) when the total score was 8-9. Cancer staging was decided by the TNM system of the American Joint Committee on Cancer (AJCC) **(8^th^ edition)** (22).

### Controlled Ovarian Stimulation Protocol

All cycles (n = 91) were conducted using the letrozole-combined GnRH antagonist protocol on the day of initial visit, without consideration of the menstrual cycle day (23). Ovarian stimulation was performed with either recombinant follicle stimulating hormone (rFSH; Gonal-F^®^; Merck Serono, Geneva, Switzerland) or FSH mixed with luteinizing hormone (LH) (FSH+LH; IVF-M, LG Chem, Korea). The starting dose of gonadotropin was administered according to the ovarian reserve and age. When the leading follicle reached a mean diameter of 13-14 mm, a GnRH antagonist (Cetrotide, 0.25 mg; Serono, Darmstadt, Germany) was added to inhibit a premature LH surge. When two or more leading follicles reached a mean diameter of ≥19 mm, the final oocyte maturation was triggered using one of the following triggering agents: 250 μg or 500 μg of recombinant human chorionic gonadotropin (rhCG; Ovidrel^®^; Merck-Serono, Darmstadt, Germany), GnRH agonist (0.2 mg of triptorelin; Decapeptyl^®^; Ferring Korea), or combination of 250 μg of rhCG and a GnRH agonist. At 36 h after the trigger, oocyte retrieval was conducted under transvaginal ultrasonographic guidance. Developmental status of the oocytes was assessed by two skilled embryologists.

**Figure 1:**
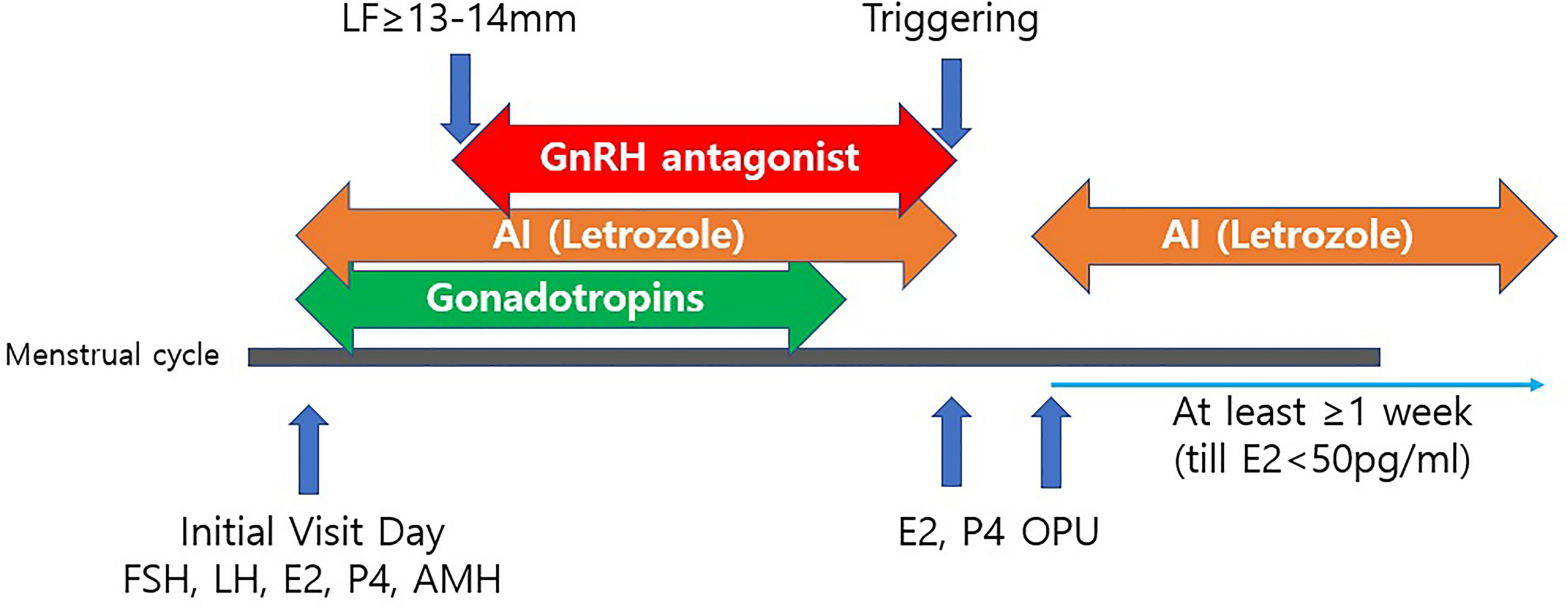
A controlled ovarian stimulation schedule for patients with breast cancer. LF, leading follicle; AI, Aromatase Inhibitor (Letrozole 5mg/day); Triggering with either rhCG, GnRH agonist or dual triggering; OPU, Ovum Pick-Up; rhCG, recombinant human chorionic gonadotropin; GnRH, Gonadotropin releasing hormone; FSH, follicle-stimulating hormone; LH, Luteinizing hormone; E2, Estradiol; P4, Progesterone; AMH, Anti-Müllerian hormone.

Five milligrams of letrozole (Novartis, Switzerland) were co-administered with gonadotropin from the start of the cycle and continued until the trigger day. Letrozole was skipped the day after the trigger and resumed after oocyte retrieval. The drug was continued for at least one week until the serum estradiol (E2) levels remained lower than 50 pg/ml (24). A COS schedule for patients with breast cancer is presented in **Figure 1**.

### Blood Sample Analysis

Peripheral blood samples were collected in the time period between the diagnosis and initiation of COS cycles. The number of WBCs was determined using a hemocytometer. Percentages of different cell types in the CBC were obtained using an automated hematology analyzer XN series (SYSMEX CORPORATION, JAPAN) for all patients before COS. All measurements and processes were conducted according to the standard laboratory manual. Most of the measurements were obtained within 1 month before COS, and all values were obtained within 2 months. MPV was directly measured in the CBC while the MPV/PC ratio was calculated by dividing MPV by the platelet count (10^4^/µL). NLR was calculated by dividing the absolute neutrophil count by the absolute lymphocyte count. PLR was calculated by dividing the absolute platelet count by the absolute lymphocyte count, and LMR was determined by dividing the absolute lymphocyte count by the absolute monocyte count.

### Cycle Outcomes

Primary outcomes included the total number of retrieved oocytes and the number of mature (metaphase II, MII) oocytes. The incidence of ovarian hyperstimulation syndrome (OHSS) was reported as a secondary outcome. The presence of OHSS was defined as a case classified as mild OHSS or higher (25). Other cycle outcome parameters, such as duration of stimulation, and total doses of gonadotropin were also compared.

### Ethics Statement

The study was approved by the Institutional Review Board (IRB) of the Seoul National University Bundang Hospital (SNUBH) (IRB No. B-2109-706-105).

### Statistical Analysis

All statistical analyses were performed using the SPSS package version 25.0 (SPSS Inc., Chicago, IL, USA). Receiver operating characteristic (ROC) curve analysis was performed to identify the optimal cutoff values of MPV and other blood markers to stratify favorable COS outcomes. Area under the ROC curve (AUC) was calculated as a measure of the power of each potential marker to discriminate between the two groups. For meaningful blood markers with statistically significant ROC curves, association between each blood marker and each clinicopathological variable, and significance of the same, was evaluated. Continuous variables are presented as mean ± SD (95% CI) using Student’s t-test to compare the variance across groups. For categorical variables, numbers were provided in percentages, and the chi-squared test or Fisher’s exact test was used as appropriate. For correlation analysis between variables, Pearson correlation analysis was used. Statistical significance was set at p < 0.05. Logistic regression analysis was performed to evaluate the association between dependent (acquisition of 10 or more MII oocytes) and independent variables (age, body mass index (BMI), AMH, MPV, MPV≥10.15, MPV/PC, MPV/PC≥0.41, NLR, PLR, and LMR).

## Results

**Figure 2:**
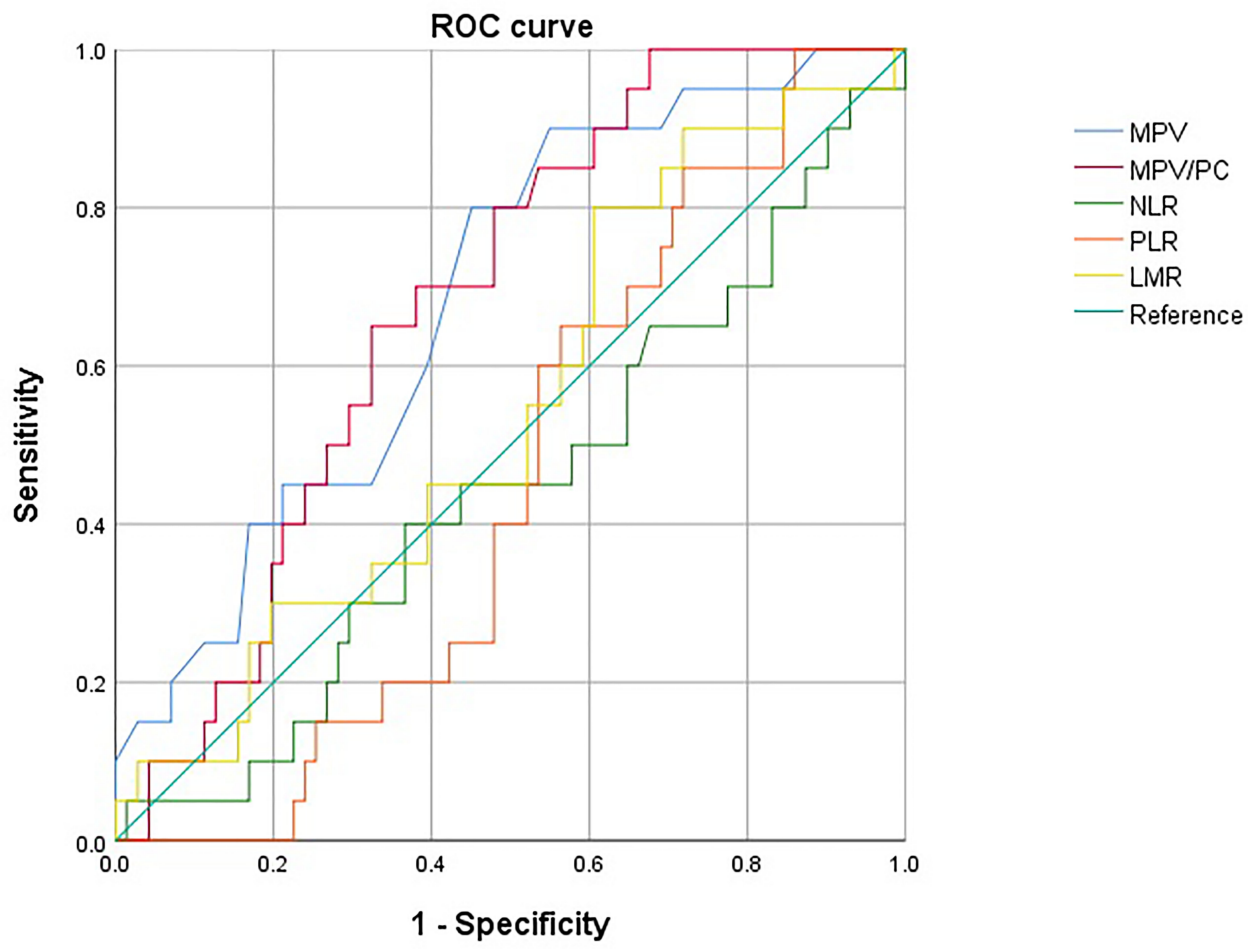
Receiver operating characteristic (ROC) curves of MPV, MPV/PC, NLR, PLR, and LMR. The optimal cutoff levels for MPV and MPV/PC were 10.15 [sensitivity: 90.0%, specificity: 45.1%, Area under the curve (AUC): 0.687, 95% CI (0.563, 0.810)] and 0.41 [sensitivity: 65.0%, specificity: 67.6%, Area under the curve (AUC): 0.682, 95% CI (0.568, 0.796)], respectively. MPV, Mean platelet volume; MPV/PC, Mean platelet volume-to-platelet count ratio; NLR, Neutrophil-to-lymphocyte ratio; PLR, Platelet-to-lymphocyte ratio; LMR, Lymphocyte-to-monocyte ratio.

In total, 91 patients with breast cancer were enrolled in this study, and their baseline demographic, clinical, and laboratory characteristics were analyzed. We conducted ROC analysis to set the optimal cutoff values for various prognostic markers to predict COS outcomes (**Table 1** and **Figure 2**). For each indicator, we set a cutoff value corresponding to the acquisition of 10 or more MII oocytes during COS. Some studies have suggested patients to freeze at least 10–20 oocytes based on age, although there are limited concrete data to support recommendations on the ideal number of oocytes to store. A recent study found oocyte vitrification to be an efficient option for elective FP when at least 8-10 MII oocytes are collected; the approach has yielded reasonable success (26, 27). Based on these criteria, a good prognosis was assigned to the group including 10 or more MII oocytes, and ROC was analyzed accordingly.

**Table 1:** Receiver operating characteristic (ROC) curve analysis of prognostic markers predicting favorable COS outcomes.

	Cutoff	AUC	Sensitivity (%)	Specificity (%)	95% Confidence Interval	p-value
MPV	10.15	0.687	90.0	45.1	[0.563, 0.810]	0.011
MPV/PC	0.41	0.682	65.0	67.6	[0.568, 0.796]	0.013
NLR	6.04	0.445	5.0	98.6	[0.301, 0.588]	0.452
PLR	97.3	0.454	100	14.1	[0.333, 0.576]	0.533
LMR	4.26	0.546	80.0	39.4	[0.408, 0.684]	0.533

COS, Controlled ovarian stimulation; AUC, Area under the curve; CI, Confidence interval; MPV, Mean platelet volume; MPV/PC, Mean platelet volume-to-platelet count ratio; NLR, Neutrophil-to-lymphocyte ratio; PLR, Platelet-to-lymphocyte ratio; LMR, Lymphocyte-to-monocyte ratio.

The optimal cutoff levels for MPV and MPV/PC were 10.15 (sensitivity: 90.0%, specificity: 45.1%, AUC: 0.687, 95% confidence Interval (CI) [0.563, 0.810]) and 0.41 (sensitivity: 65.0%, specificity: 67.6%, AUC: 0.682, 95% CI [0.568, 0.796]), respectively. Unlike MPV and MPV/PC, NLR, PLR, and LMR did not show any statistically significant difference in relation to their cutoff values. Only the parameters showing a statistical significance (i.e., MPV and MPV/PC) were evaluated further.

To compare the usefulness of MPV and MPV/PC at the set cutoff values in predicting COS outcomes, we compared the basal characteristics and COS-related parameters. At the MPV cutoff value of 10.15, there was no differences in age, BMI, and AMH levels between the two groups. There was no difference between the two groups in terms of cancer staging, grade, or receptor positivity (**Table 2**). However, the total number of oocytes obtained, and the number of MII oocytes were significantly lower in the group with MPV<10.15 than in the group with MPV≥10.15 (8.0 ± 5.1 *vs.* 12.6 ± 9.1, p = 0.003; 4.0 ± 3.7 *vs.* 7.3 ± 6.3, p = 0.002, respectively). The frequency of OHSS did not differ between the two groups (**Table 3**).

**Table 2:** Basal characteristics of patients with breast cancer at the MPV cutoff value 10.15 (n=91).

	MPV <10.15 (n=32)	MPV ≥10.15 (n=59)	p-value
Age (years)	33.5 ± 5.7	33.7 ± 4.8	0.874
BMI (Kg/m^2^)	22.5 ± 3.7	21.9 ± 3.0	0.378
AMH (ng/ml)	3.3 ± 3.2	3.9 ± 3.3	0.435
Breast cancer type			0.162
DCIS	3 (9.3%)	3 (5.1%)	
IDC	25 (78.1%)	54 (91.5%)	
Metaplastic	0 (0%)	1 (1.7%)	
Mucinous	2 (6.3%)	1 (1.7%)	
Others*	2 (6.3%)	0 (0%)	
Histologic grade**			0.256
1	7 (7/30, 23.3%)	7 (7/58, 12.1%)	
2	9 (9/30, 30.0%)	26 (26/58, 44.8%)	
3	14 (14/30, 46.7%)	25 (25/58, 43.1%)	
Stage			0.212
0	0 (0%)	2 (3.4%)	
1	18 (56.2%)	26 (44.1%)	
2	6 (18.7%)	11 (18.6%)	
3	4 (12.5%)	18 (30.5%)	
4	2 (6.3%)	1 (1.7%)	
unknown	2 (6.3%)	1 (1.7%)	
ER status			0.137
Positive	21 (65.6%)	44 (74.6%)	
Negative	9 (28.1%)	15 (25.4%)	
Unknown	2 (6.3%)	0 (0%)	
PR status			0.133
Positive	20 (62.5%)	36 (61.0%)	
Negative	10 (31.3%)	23 (39.0%)	
Unknown	2 (6.2%)	0 (0%)	
HER2 status			0.066
Positive	15 (46.9%)	38 (64.4%)	
Negative	15 (46.9%)	21 (35.6%)	
Unknown	2 (6.2%)	0 (0%)	
Triple negative breast cancer (TNBC)			0.439
Yes	6 (18.7%)	9 (15.3%)	
No	26 (81.3%)	50 (84.7%)	
Initial CA 15-3	10.3 ± 4.1	10.1 ± 5.5	0.840

**-** Data are shown as either Mean ± SD or N (%).

BMI, Body Mass Index; AMH, Anti-Müllerian hormone; DCIS, Ductal carcinoma in situ; IDC, Invasive ductal carcinoma; ER, Estrogen receptor; PR, Progesterone receptor.

*Others: 1 malignant Phyllodes tumor, 1 mixture of IDC and Acinic cell carcinoma.

** Histologic grade: grade 1 (well differentiated), grade 2 (moderately differentiated), grade 3 (poorly differentiated).

HER2: Human epidermal growth factor receptor 2.

**Table 3:** Controlled ovarian stimulation outcomes of patients with breast cancer at the MPV cutoff value 10.15 (n=91).

	MPV <10.15 (n=32)	MPV ≥10.15 (n=59)	p-value
Days of stimulation (days)	8.6 ± 1.7	8.9 ± 2.0	0.485
Total dose of gonadotropins (IUs)	2308.6 ± 648.2	2323.7 ± 695.7	0.919
Triggering agent (n, %)			0.182
rhCG	19 (59.3%)	24 (40.7%)	
Dual triggering	11 (34.4%)	26 (44.0%)	
GnRH agonist	2 (6.3%)	9 (15.3%)	
Stimulation start day			0.764
Follicular	16 (50.0%)	28 (47.5%)	
Luteal	15 (46.9%)	27 (45.8%)	
unknown	1 (3.1%)	4 (6.8%)	
Peak E2 at triggering day (pg/ml)	412.0 ± 318.2	430.7 ± 329.8	0.812
Total retrieved oocytes (n)	8.0 ± 5.1	12.6 ± 9.1	0.003
Mature (MII) oocytes (n)	4.0 ± 3.7	7.3 ± 6.3	0.002
Maturation rate (%)	47.5 ± 30.0	53.5 ± 28.3	0.343
OHSS			0.233
No	29 (90.6%)	57 (96.6%)	
Yes	3 (9.4%)	2 (3.4%)	

- Data are shown as either Mean ± SD or N (%).

rhCG, recombinant human chorionic gonadotropin; GnRH, gonadotropin-releasing hormone; Dual triggering, rhCG+GnRH agonist; E2, Estradiol; OHSS, ovarian hyperstimulation syndrome.

At the MPV/PC cutoff value of 0.41, there was no differences in basal characteristics, the amount of gonadotropin used for COS, and the duration of stimulation between the low- and high-MPV/PC groups (**Tables 4**, **5**). Meanwhile, the total number of oocytes obtained was significantly lower in the low-MPV/PC group than in the high-MPV/PC group (9.5 ± 7.1 *vs.* 13.1 ± 9.1, p = 0.048). The number of mature oocytes also showed similar results, but the difference was not statistically significant (5.3 ± 5.4 *vs.* 7.3 ± 6.1, p = 0.092).

**Table 4:** Basal characteristics of patients with breast cancer at the MPV/PC cutoff value 0.41 (n=91).

	MPV/PC <0.41 (n=53)	MPV/PC ≥0.41 (n=38)	p-value
Age (years)	34.3 ± 5.2	32.6 ± 4.7	0.108
BMI (Kg/m^2^)	22.7 ± 3.3	21.4 ± 3.1	0.067
AMH (ng/ml)	4.2 ± 3.9	3.1 ± 2.1	0.090
Breast cancer type			0.208
DCIS	2 (3.8%)	4 (10.5%)	
IDC	45 (84.9%)	34 (89.5%)	
Metaplastic	1 (1.9%)	0 (0%)	
Mucinous	3 (5.7%)	0 (0%)	
Others^*^	2 (3.7%)	0 (0%)	
Histologic grade**			0.543
1	8 (8/51, 15.7%)	6 (6/37, 16.2%)	
2	18 (18/51, 35.3%)	17 (17/37, 45.9%)	
3	25 (25/51, 49.0%)	14 (14/37, 37.9%)	
Stage			0.926
0	1 (1.8%)	1 (2.6%)	
1	26 (49.1%)	18 (47.4%)	
2	8 (15.1%)	9 (23.7%)	
3	14 (26.4%)	8 (21.1%)	
4	2 (3.8%)	1 (2.6%)	
unknown	2 (3.8%)	1 (2.6%)	
ER status			0.972
Positive	38 (71.7%)	27 (71.1%)	
Negative	14 (26.4%)	10 (26.3%)	
Unknown	1 (1.9%)	1 (2.6%)	
PR status			0.964
Positive	33 (62.3%)	23 (60.6%)	
Negative	19 (35.8%)	14 (36.8%)	
Unknown	1 (1.9%)	1 (2.6%)	
HER2 status			0.889
Positive	30 (56.6%)	23 (60.6%)	
Negative	22 (41.5%)	14 (36.8%)	
Unknown	1 (1.9%)	1 (2.6%)	
Triple negative breast cancer (TNBC)			0.777
Yes	8 (15.1%)	7 (18.4%)	
No	45 (84.9%)	31 (81.6%)	
Initial CA 15-3	11.1 ± 5.6	8.9 ± 3.9	0.050

**-** Data are shown as either Mean ± SD or N (%).

BMI, Body Mass Index; AMH, Anti-Müllerian hormone; DCIS, Ductal carcinoma in situ; IDC, Invasive ductal carcinoma; ER, Estrogen receptor; PR, Progesterone receptor.

*Others: 1 malignant Phyllodes tumor, 1 mixture of IDC and Acinic cell carcinoma.

**Histologic grade: grade 1 (well differentiated), grade 2 (moderately differentiated), grade 3 (poorly differentiated).

HER2, Human epidermal growth factor receptor 2.

**Table 5:** Controlled ovarian stimulation outcomes of patients with breast cancer at the MPV/PC cutoff value 0.41 (n=91).

	MPV/PC <0.41 (n=53)	MPV/PC ≥0.41 (n=38)	p-value
Days of stimulation (days)	8.9 ± 1.9	8.6 ± 1.9	0.438
Total dose of gonadotropins (IUs)	2336.3 ± 749.9	2293.4 ± 565.2	0.767
Total letrozole dose till triggering day (mg)	49.3 ± 11.4	48.6 ± 9.4	0.728
Triggering agent (n, %)			0.095
rhCG	30 (56.6%)	13 (34.2%)	
Dual triggering	17 (32.1%)	20 (52.6%)	
GnRH agonist	6 (11.3%)	5 (13.2%)	
Stimulation start day			0.397
Follicular	23 (43.4%)	21 (55.3%)	
Luteal	26 (49.1%)	16 (42.1%)	
unknown	4 (7.5%)	1 (2.6%)	
Peak E2 at triggering day (pg/ml)	405.3 ± 286.7	450.0 ± 370.9	0.547
Total retrieved oocytes (n)	9.5 ± 7.1	13.1 ± 9.1	0.048
Mature (MII) oocytes (n)	5.3 ± 5.4	7.3 ± 6.1	0.092
Maturation rate (%)	49.1 ± 30.3	54.6 ± 26.7	0.368
OHSS			0.655
No	50 (94.3%)	36 (94.7%)	
Yes	3 (5.7%)	2 (5.3%)	

- Data are shown as either Mean ± SD or N (%).

rhCG, recombinant human chorionic gonadotropin; GnRH, gonadotropin-releasing hormone; Dual triggering, rhCG+GnRH agonist; E2, Estradiol; OHSS, ovarian hyperstimulation syndrome.

**Figure 3:**
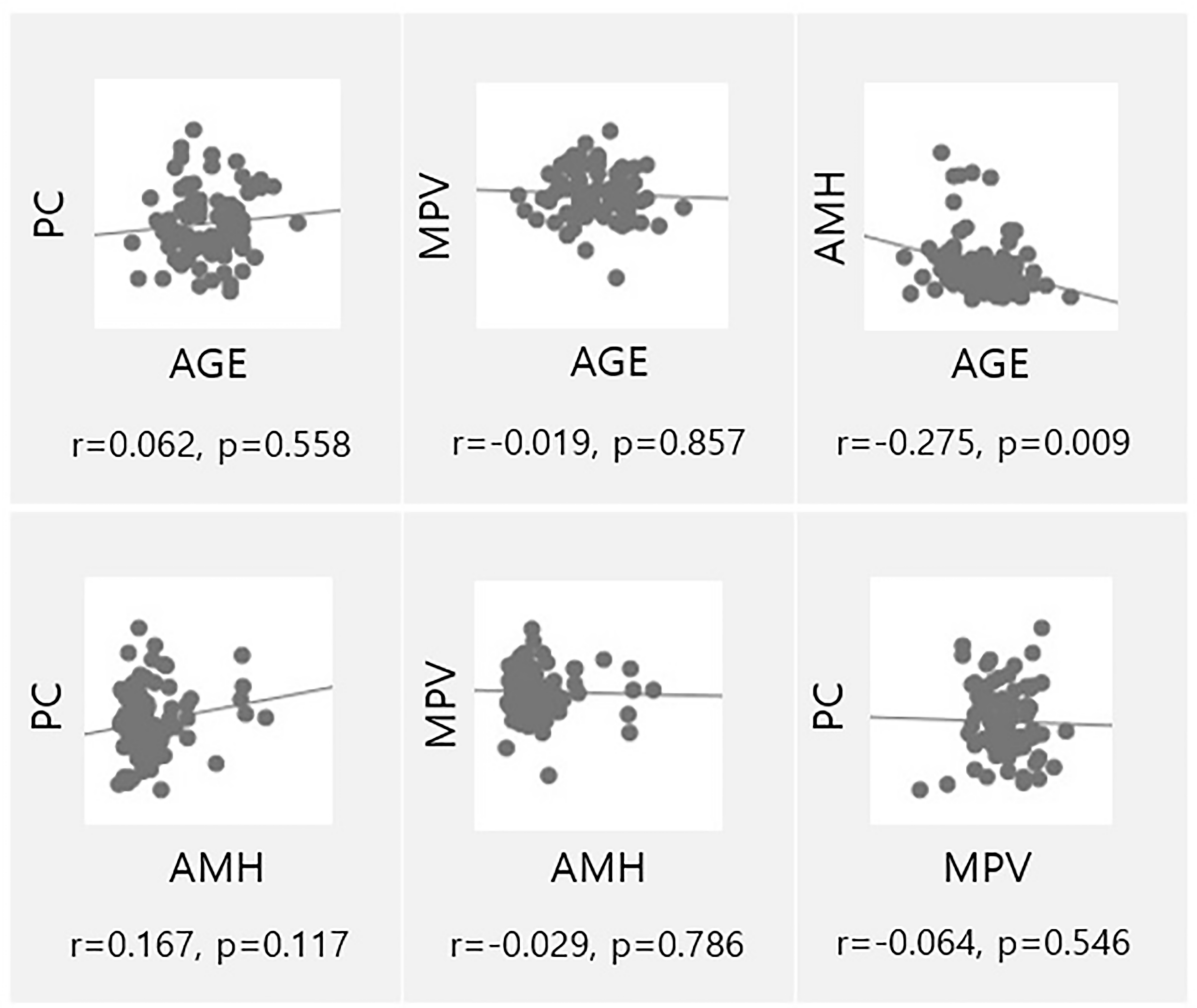
Univariate analysis of ovarian reserve markers (AMH, Age) and parameters associated to platelets (MPV, PC). PC, Platelet count; AMH, Anti-müllerian hormone; MPV, Mean platelet volume.

Age and AMH levels are currently used as markers of ovarian function. Correlation analysis was performed to analyze the associations across platelet parameters, age, and AMH levels (**Figure 3**). As expected, age and AMH levels were significantly correlated (p=0.009), but no correlations were observed for the other parameters.

For logistic regression analysis, age, BMI, AMH, MPV, MPV≥10.15, MPV/PC, MPV/PC≥0.41, NLR, PLR, and LMR were set as independent variables to analyze the dependent variables (acquisition of 10 or more MII oocytes). AMH, MPV, and MPV/PC≥0.41 were positive predictive markers (p=0.000, OR: 1.622; p=0.018, OR: 3.184; p=0.013, OR: 9.251, respectively) and age was a negative predictive marker (p=0.049, OR: 0.850) for 10 or more MII oocyte acquisition. **Table 6** shows the details.

**Table 6:** Logistic regression analysis of factors affecting acquisition of 10 or more MII oocytes.

	p-value	Odds ratio	95% Confidence Interval
Age	0.049	0.850	[0.723, 0.999]
AMH	0.000	1.622	[1.236, 2.126]
MPV	0.018	3.184	[1.215, 8.339]
MPV/PC≥0.41	0.013	9.251	[1.612, 53.093]

Independent variables, Age, BMI, AMH, MPV, MPV≥10.15, MPV/PC, MPV/PC≥0.41, NLR, PLR, LMR.

MII, metaphase II; AMH, Anti-Müllerian hormone; BMI, Body mass index; MPV, Mean platelet volume; MPV/PC, Mean platelet volume-to-platelet count ratio; NLR, Neutrophil-to-lymphocyte ratio; PLR, Platelet-to-lymphocyte ratio; LMR, Lymphocyte-to-monocyte ratio.

## Discussion

Here, we demonstrated the effectiveness of blood markers for predicting COS outcomes in patients with breast cancer, and the findings indicated that MPV and MPV/PC could be used as markers in clinical practice. Our results showed that patients with MPV<10.15 had worse COS outcomes with low total and MII oocyte acquisition than those with MPV≥10.15. Similar results were seen in patients with MPV/PC<0.41 than in those with MPV/PC ≥ 0.41. Age, AMH, MPV, and MPV/PC were significant factors in the acquisition of 10 or more MII oocytes. Together with conventional ovarian reserve markers, current findings can be used to develop patient-tailored strategies, wherein methods such as the dual-trigger approach can be used to increase maturity in the group that is expected to show a low mature oocyte yield and a high dose of gonadotropin can be considered for the group that is expected to show a low total oocyte yield.

Inflammation and cancer are strongly associated; pretreatment levels of peripheral inflammatory cells, including neutrophils, lymphocytes, and monocytes, have been reported to serve as prognostic factors in various cancers (28). Attempts to reveal the appropriate cut-off values of inflammatory markers linked with the prognosis of breast cancer had been investigated by ROC curve analyses in many studies; however, the authors failed to reach a consensus (29). In our study, low MPV and high platelet counts were both associated with poor COS outcomes. Although no previous study had examined the relationship of MPV or the platelet count with COS outcomes, low MPV or high platelet count had been reported to be associated with a poor prognosis and metastasis in patients with breast cancer (30, 31). MPV indicates the platelet size, and an increase in this value is an indicator of a large number of reactive platelets resulting from increased platelet turnover. MPV also reflects the level of platelet stimulation and the rate of platelet production.

Low oocyte maturity in low-MPV groups has been postulated to be attributable to a lack of or insufficiency of platelet reactivity. During low-grade inflammation, as the platelet size increases, enzymatic activity is increased and a greater number of cytokines are released into the environment (32). Platelets are directly involved in ovarian function. Fujiwara et al. showed that a considerable number of platelets and red blood cells were present at extravascular sites in luteinizing granulosa cells after ovulation (33). In a study using immature rats, platelets were activated by platelet activating factor (PAF) produced in the ovary during gonadotropin-induced ovulation, and PAF was involved in follicle rupture (34). A recent review by Bódis et al. indicated that platelets modulate the function of the hypothalamo-hypophyseal-ovarian system (35). Specifically, hypothalamic GnRH induces FSH from the anterior pituitary, which in turn induces and stimulates follicular and oocyte maturation and steroid hormone secretion in the ovary. Simultaneously, follicular cells enhance PAF production. Through these pathways, activated platelets accumulate in the follicular vessels surrounding the follicle (36), and through the release of various kinds of soluble molecules in their granules (factors, mediators, chemokines, cytokines, and neurotransmitters), the platelets locally increase oocyte maturation and hormone secretion. Platelets also contain multiple adhesion molecules and receptors on their surfaces. Brain-derived neurotrophic factor (BDNF), which is released mainly from the brain, may also contribute to the maintenance of ovarian homeostasis, emphasizing the importance of BDNF in the paracrine regulation of ovarian function (37). BDNF has been identified in oocytes and follicular cells and is reported to be related to follicle development, oocyte maturation, and survival (38, 39); BDNF receptors have been found on the surface of these cells. Furthermore, BDNF can be visualized in proplatelets as well, which suggested that platelets already contain BDNF by the time they begin to separate from megakaryocytes (40). Bódis et al. designated this system as a “platelet-associated regulatory system (PARS)”. In summary, signal (PAF) from the ovary activates platelets and the release of various platelet factors. Among the platelet factors, BDNF may play a critical role in follicle development and oocyte maturation. Although the mechanism underlying platelet involvement in ovarian physiology is still unknown and should be further elucidated, disruption of platelet activation and homeostasis remains one of the possible explanations for low oocyte yields and maturity. Molecular studies would be necessary to elucidate the underlying mechanism.

Currently, AMH and AFC are used as significant indicators for predicting COS outcomes, including the ovarian response, in clinical practice. However, the host tumor environment in patients with cancer differs from that of the general population and can have systemic effects. Furthermore, mean age and AMH levels did not differ between the two groups in our study. Therefore, the availability of additional markers other than AMH and AFC to predict outcomes and allow the development of more specific and tailored protocols will be of great benefit to women with breast cancer, most of whom have only one opportunity to undergo FP before chemotherapy.

Although platelets are not directly related to ovarian reserve markers such as age or AMH, they can be employed as “functional” markers that can predict COS outcome. In patients with breast cancer at a young reproductive age, conventional ovarian reserve markers are within a narrow range; thus, blood markers can be utilized for additional information. Our findings will help clinicians consider blood markers for predicting COS outcomes and for decision-making, thereby enabling individual counseling as required. As markers based on CBC, which is routinely checked in patients with breast cancer, blood markers have the advantage of being easy, fast, and economical to use.

To the best of our knowledge, this is the first study to elucidate the association between blood inflammatory markers and COS outcomes in patients with breast cancer undergoing FP. The strength of our study is that we only included patients with breast cancer undergoing COS with a single protocol, ensuring consistency and uniformity of the results. However, the study has some limitations. Previous studies had reported several important confounders of MPV; sex, age, obesity, and duration of smoking are possible confounders affecting platelet morphology and size (32). In the present study, data for smoking history were not properly obtained due to the lack of information in the electronic medical records, which complicated the assessment of the influence of duration of smoking on MPV. Nevertheless, the mean BMI and age of the two groups according to the cutoff value were statistically similar between the groups in one sex, and those may minimize the confounding effects on MPV. Hypertension (32) and diabetes (41) can also affect platelet size, and we excluded patients with hypertension, heart failure, hepatic and renal disorders, vascular disease, and hematological diseases. The retrospective nature of the investigation is also a limitation of the study, and a prospective study recruiting many more patients will be required in future. Moreover, we only enrolled a small number of patients with breast cancer, and additional research would be required to determine whether the markers have predictive power in a large number of patients or patients with other types of cancer. This study only included in parameters related to CBC, due to which, systemic inflammation markers, such as C-reactive protein, interleukins, and tumor necrosis factor-α were not evaluated.

In conclusion, we verified the effectiveness of MPV and MPV/PC as new prognostic markers that can be used for predicting COS outcomes and establishing individualized FP strategies for patients with breast cancer. The new predictors can be expected to maximize FP outcomes in patients with limited opportunities for FP.

## Data Availability Statement

The raw data supporting the conclusions of this article will be available on reasonable request to the corresponding author.

## Ethics Statement

The studies involving human participants were reviewed and approved by Institutional Review Board (IRB) of the Seoul National University Bundang Hospital (IRB No. B-2109-706-105). Written informed consent for participation was not required for this study in accordance with the national legislation and the institutional requirements.

## Author Contributions

YH and JL: design of the study and the manuscript writing. YH, SK, JL, and CS: gathering and analyzing the data. All authors: discussion of the results and revision of the manuscript. All authors contributed to the article and approved the submitted version.

## Conflict of Interest

The authors declare that the research was conducted in the absence of any commercial or financial relationships that could be construed as a potential conflict of interest.

## Publisher’s Note

All claims expressed in this article are solely those of the authors and do not necessarily represent those of their affiliated organizations, or those of the publisher, the editors and the reviewers. Any product that may be evaluated in this article, or claim that may be made by its manufacturer, is not guaranteed or endorsed by the publisher.
